# Supplementary material for: Suppression of Breast Cancer by Small Molecules That Block the Prolactin Receptor
Source: Cancers (Basel). 2021 May 28;13(11):2662. doi: 10.3390/cancers13112662 (PMC8198871; doi:10.3390/cancers13112662)
Supplement: Supplementary file 1 [file cancers-13-02662-s001.zip › cancers-1230698-supplementary.pdf]

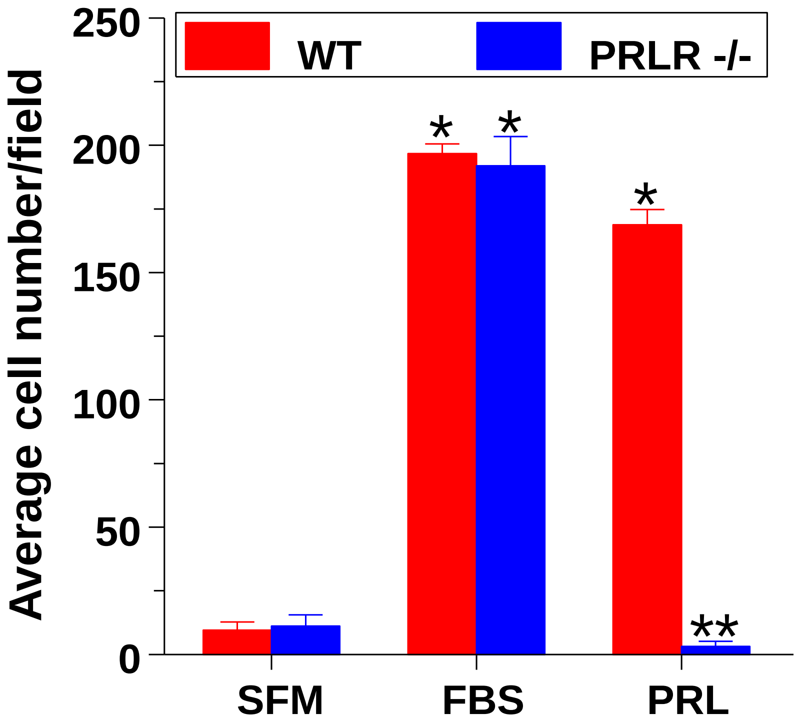

Figure S1: Stimulation of invasion of MDA-MB-468 cells by PRL and lack of responsiveness to PRL in cells with inactivated PRLR ($PRLR^{-/-}$). Cells plated in Boyden chambers with porous membranes were treated with SFM (10% serum free medium), FBS (10% fetal bovine serum), or PRL (1nM). After 24 hrs, invading cells on the membrane underside were counted under the microscope. Values are means ± SEM (n= 3). * significant ($p<0.05$) vs SFM; ** significant vs PRL.

SMI-1

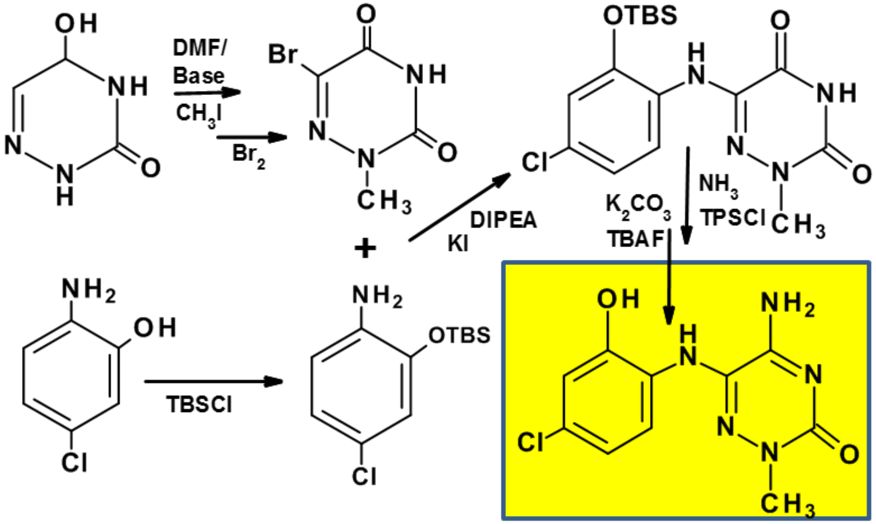

SMI-6

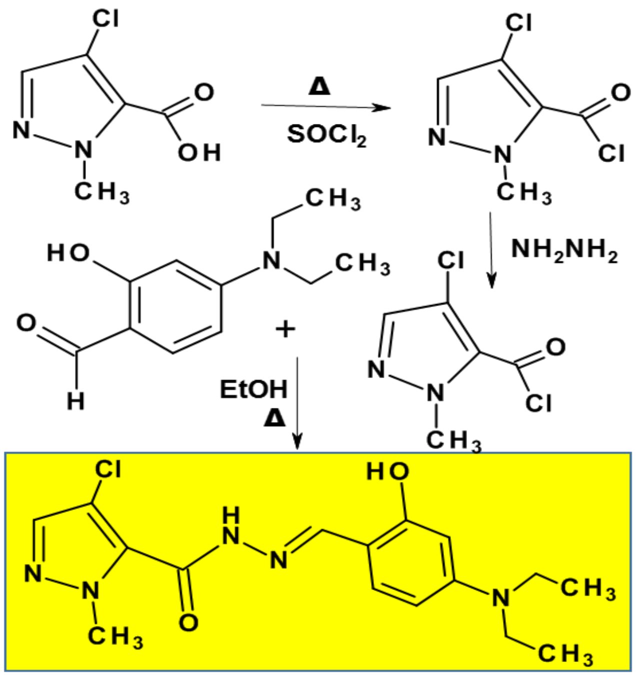

Figure S2: Steps in the biosynthesis of SMI-1 and SMI-6

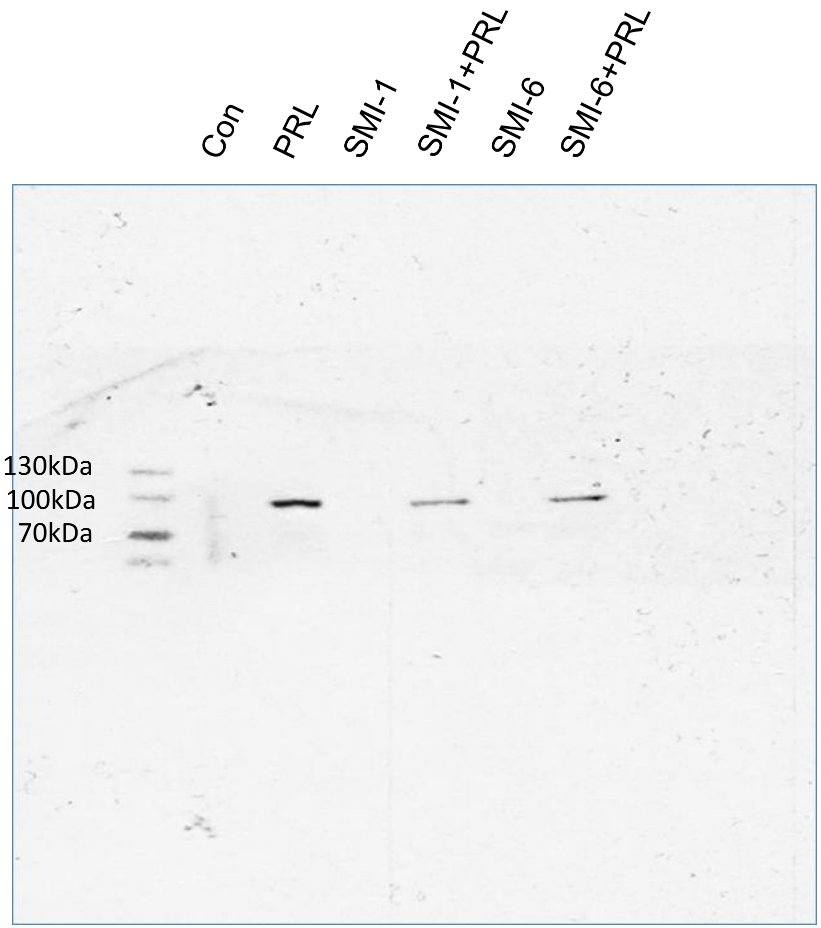

Figure S3: Full blot Phospho-Jak2

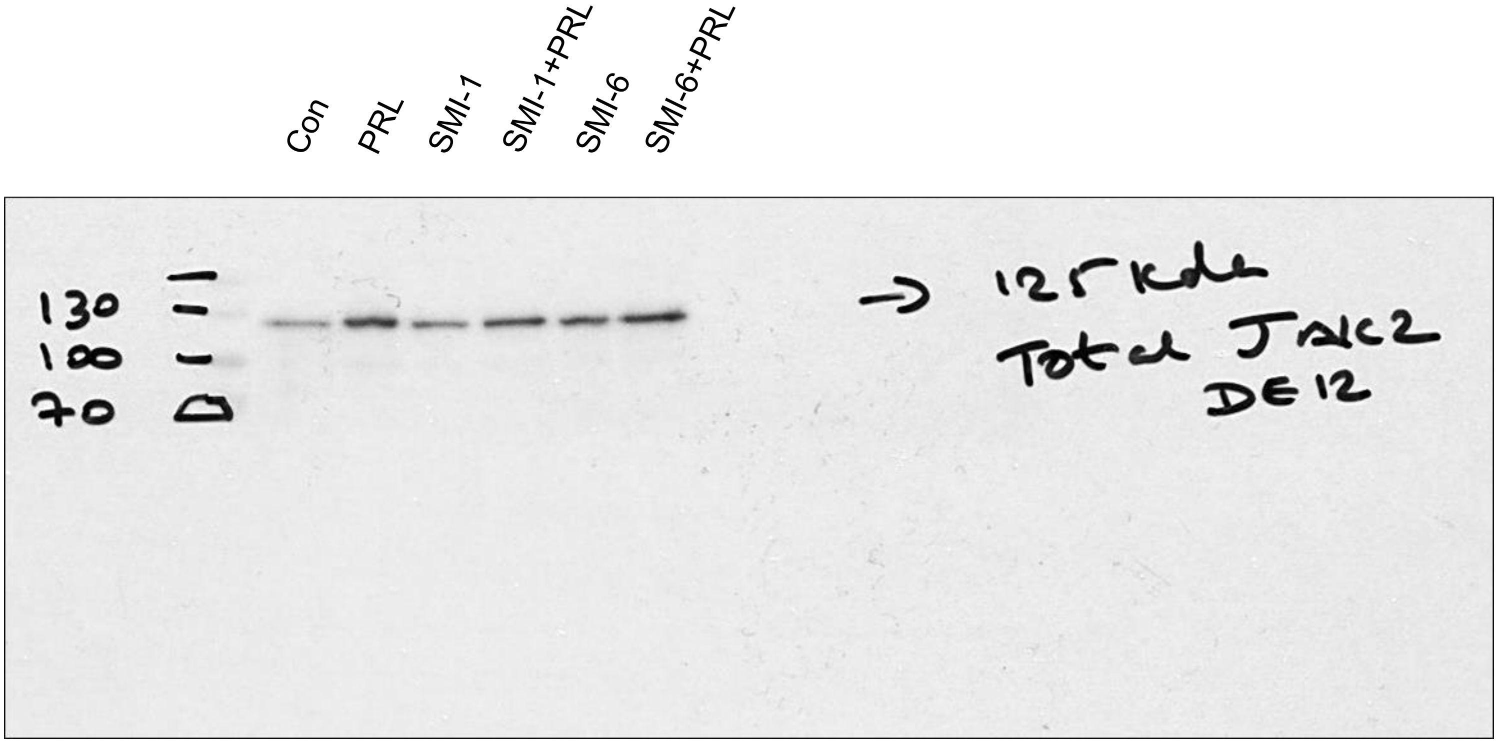

Figure S4: full blot Total Jak2
